# Neighborhood playability and early childhood development: a population-based birth cohort study

**DOI:** 10.1016/j.envres.2026.124124

**Published:** 2026-03-03

**Authors:** Ingrid Jarvis, Emily Gemmell, Alicia Cavanaugh, Zoë Davis, Martin Guhn, Hind Sbihi, Tim F. Oberlander, Matilda van den Bosch, Michael Brauer

**Affiliations:** a School of Population and Public Health, University of British Columbia, 2206 East Mall, Vancouver, British Columbia, Canada; b Department of Geography, McGill University, 805 Sherbrooke Street West, Montreal, Quebec, Canada; c Department of Epidemiology, Biostatistics and Occupational Health, McGill University, 2001 McGill College, Montreal, Quebec, Canada; d School of Agriculture, Food and Ecosystem Sciences, University of Melbourne, 500 Yarra Blvd, Richmond, Victoria, 3121, Australia; e Human Early Learning Partnership, School of Population and Public Health, University of British Columbia, 2206 East Mall, Vancouver, British Columbia, Canada; f British Columbia Centre for Disease Control, 655 W 12th Ave, Vancouver, British Columbia, Canada; g Department of Pediatrics, Faculty of Medicine, University of British Columbia, 4480 Oak Street, Vancouver, British Columbia, Canada; h ISGlobal, Parc de Recerca Biomèdica de Barcelona, Doctor Aiguader 88, 08003, Barcelona, Spain; i Universitat Pompeu Fabra, Plaça de la Mercè, 10-12, 08002, Barcelona, Spain; j CIBER Epidemiología y Salud Pública, Av. Monforte de Lemos, 3-5, 28029, Madrid, Spain; k The European Forest Institute, Biocities Facility, Via Manziana 30, 00189, Rome, Italy

**Keywords:** built environment, neighborhood, environmental health, early child development, outdoor free play

## Abstract

**Background:**

Outdoor play is important for children’s health and development. Research indicates that neighborhood characteristics influence children’s engagement in outdoor play, but no studies have examined associations between neighborhood playability and childhood development.

**Methods:**

Using a population-based child cohort (n=30,126) in Metro Vancouver, Canada, we studied the association between early-life neighborhood playability and early childhood development. Neighborhood playability, the suitability of an area for young children to engage in outdoor free play, and its five domains (traffic environment, spaces for play, social environment, natural environment, child-relevant destinations) was quantified with a novel geospatial metric. Early childhood development was assessed via teacher-ratings on the Early Development Instrument that measures children’s development in physical, social, emotional, and cognitive domains (mean age = 5.6 years). Multilevel models were used to analyze the association between neighborhood playability, as measured by a composite score and individual domain scores, and early childhood development.

**Results:**

Following adjustment for demographic and socio-economic variables, the highest quartile of composite neighborhood playability was associated with higher child development scores (b: 0.30, 95% CI: 0.05, 0.54) and lower odds of developmental vulnerability (OR: 0.90; 95% CI: 0.82, 0.98). Associations varied across playability domain scores, with the strongest positive association observed for the natural environment domain, followed by the social and traffic environment domains. Negative associations were observed for the child-relevant destinations domain score.

**Conclusions:**

The results suggest that increased neighborhood playability, particularly natural environments, may promote healthy early childhood development. Findings inform urban planning policies and interventions that support childhood development.

## 1 Introduction

Early-life experiences are important for shaping human development and lifelong health trajectories (Gluckman et al., 2008). Outdoor play is increasingly recognised for the physical, social, emotional, and cognitive health and developmental benefits it affords children (Brussoni et al., 2015; Dankiw et al., 2020; Gibson et al., 2017; Gray et al., 2015). The fundamental importance of play to children’s well-being has been codified in the United Nations Convention on the Rights of the Child (Office of the United Nations High Commissioner for Human Rights, 1989).

Interactions between an individual and their surrounding environment are important influences on human development and health-promoting behaviors, like outdoor play (Bronfenbrenner, 1979). Characteristics of the surrounding environment may facilitate or hinder children’s opportunities for and engagement in outdoor play, with the suitability of the neighborhood environment for outdoor play described as playability (i.e., “how friendly environments are for outdoor play and independent mobility”) (Han et al., 2018, p. 197). Though playability may be associated with more play, it is not a measure of play itself (Gemmell et al., 2026). Recent systematic reviews have identified neighborhood physical characteristics associated with more outdoor play time among children, including available home yard space, play spaces, parks and playgrounds, green space, education and recreation destinations, low traffic streets, and pedestrian-friendly walking and cycling paths (Gemmell et al., 2022; Lambert et al., 2019; E.-Y. Lee et al., 2021; Visser & van Aalst, 2022). Neighborhood social characteristics, including the presence of other children, parental trust in neighbors, parental perception of social safety, and opportunities for neighborhood social interactions, have also been positively associated with outdoor play (Andrews et al., 2019; Hunter et al., 2022; Parent et al., 2021).

Although the benefits of outdoor play to children’s health and development are well-documented (Brussoni et al., 2015; Dankiw et al., 2020; Gibson et al., 2017; Gray et al., 2015), and the neighborhood characteristics that support outdoor play have been identified (Gemmell et al., 2022; Lambert et al., 2019; E.-Y. Lee et al., 2021; Visser & van Aalst, 2022), no known studies have previously examined associations between neighborhood playability and early childhood development. Prior research has studied the influence of individual neighborhood characteristics on childhood health and development (Christian et al., 2017; Collyer et al., 2022; Turrell et al., 2020; Villanueva et al., 2023), but no composite measure of neighborhood characteristics supportive of outdoor play has been applied in this research context. In addition, research findings on the association between individual neighborhood characteristics and early childhood health and development are mixed and, except for a limited number of studies (Kronaizl & Koss, 2023; Mygind et al., 2022), prior research has not explicitly considered play as a potential pathway underlying this relationship.

We leveraged the recent development of a novel metric of neighborhood playability (Gemmell et al., 2026) to evaluate the associations between neighborhood playability and its five individual domains (i.e., traffic environment, spaces for play, social environment, natural environment, child-relevant destinations) with early childhood development in a population-based birth cohort in Metro Vancouver, Canada. Given that early childhood development and opportunities for outdoor play may vary by sex and socio-economic status (Gemmell et al., 2022; Gill et al., 2024; Talarico et al., 2023), and that children from more vulnerable groups may be more sensitive to neighborhood environmental conditions (Belsky, 2013), we assessed potential effect modification by a child’s sex and socio-economic status. We hypothesized that higher composite neighborhood playability and individual playability domain scores would be positively associated with early childhood development after adjustment for demographic and socio-economic variables. We further hypothesized these positive associations would be stronger among male children and children from lower socio-economic households, reflecting greater potential benefits from neighborhood playability in groups that may experience lower developmental outcomes (Gill et al., 2024; Talarico et al., 2023).

## 2 Materials and Methods

### 2.1 Study population

We conducted this analysis within a population-based linked administrative data birth cohort in Metro Vancouver, located in southwestern British Columbia (BC), Canada. Metro Vancouver is the most populous region in BC, housing over half of the provincial population (British Columbia BC Stats, 2025). The cohort was identified using administrative data from the BC Ministry of Health (British Columbia Ministry of Health, 2018, 2019a), the BC Vital Statistics Agency (British Columbia Ministry of Health, 2019b, 2019c), Perinatal Services BC (Perinatal Services BC, 2019), and the Human Early Learning Partnership (Human Early Learning Partnership, 2019). The Perinatal Data Registry from Perinatal Services BC contains data abstracted from obstetrical and neonatal medical records for nearly 100% of births in BC (Perinatal Services BC, 2025). It includes data from over 60 acute care facilities and home births attended by BC-registered midwives. The registry captures information on pregnancies ending in a live or still birth at ≥20 weeks’ gestation or ≥500 grams birth weight, and tracks maternal postpartum readmissions up to 42 days, and infant readmissions or transfer up to 28 days after birth. Individual data linkage of these sources was completed by Population Data BC.

The cohort comprised of all children from live, singleton births to mothers residing in Metro Vancouver from April 2000 to December 2005 and who attended kindergarten between the 2005/2006 and 2010/2011 school years. To be eligible, children and their mothers had to be registrants of the BC Medical Services Plan (MSP), a mandatory universal health insurance program in the province of BC, and to have lived in Metro Vancouver for the duration of the exposure period. In BC, MSP coverage is available to provincial residents, defined as Canadian citizens, individuals lawfully admitted for permanent residence, and certain temporary permit holders (e.g., study or work permits valid for six months or longer) who are deemed residents; short-term visitors are not eligible for coverage (Government of British Columbia, 2025). Eligible dependents of MSP beneficiaries, including children, are also covered. The exposure period was from two years of age until the time of outcome assessment, which took place in the second half of the kindergarten school year. In the province of BC, children are able to start kindergarten in September of the year they turn five years of age (Government of British Columbia, 2024). We selected two as the minimum age for the exposure period to align with the research informing the development of the playability metric (Gemmell et al., 2026). We excluded children who moved outside of the study region or had a gap in residential history (i.e., one or more years without annual six-digit postal code).

Ethics approval for our study was granted by the University of British Columbia Behavioral Research Ethics Board (certificate H18-00908). The data that underlie our study were accessed via Population Data BC with the approval of Data Stewards and terms of use were outlined in data sharing agreements that restricted the use of data for research purposes.

### 2.2 Assessment of early childhood development

We assessed early childhood development using the Early Development Instrument (EDI), a population-level tool to evaluate children’s development in kindergarten (Janus & Offord, 2007). Kindergarten teachers complete the EDI for each of their students in early spring based on classroom observations in the previous six months. The EDI comprises demographic information and 103 binary and Likert scale items assessing a child’s ability to meet age-appropriate developmental expectations in five domains: (1) physical health and well-being; (2) social competence; (3) emotional maturity; (4) language and cognitive development; and (5) communication skills and general knowledge. For each of the five domains, a child receives a domain score, calculated as the mean across all domain items, ranging from 0 (lowest) to 10 (highest). Children also receive a total EDI score, calculated as the sum of the five domain scores, ranging from 0 (lowest) to 50 (highest). Prior studies have reported on the EDI’s validity and reliability as a measure of children’s early developmental outcomes (Janus et al., 2011; Janus & Offord, 2007). For instance, studies have examined the EDI’s internal consistency across domains (Cronbach’s alpha ranging from 0.84 to 0.96) (Janus & Offord, 2007), test-retest reliability (correlations ranging from 0.82 to 0.94) (Janus & Offord, 2007), inter-rater reliability (correlations ranging from 0.53 to 0.80) (Janus & Offord, 2007), predictive validity of academic achievement in later school years (Forget-Dubois et al., 2007), and convergent validity with direct, child-based developmental assessments (Hymel et al., 2011). In the current study, we used the total EDI score as an indicator of overall early childhood development, with higher scores indicating better developmental outcomes.

### 2.3 Neighborhood playability

We assessed neighborhood playability using a recently developed, evidence-based and expert-informed geospatial playability metric that measures the suitability of urban neighbourhoods for young children’s outdoor free play (Gemmell et al., 2026). In brief, the playability metric operationalizes an evidence-based theoretical framework for urban playability (Gemmell et al., 2022). The framework focuses on identifying neighborhood features shown to influence young children’s (aged two to six years) outdoor free play across diverse contexts based on assessment of existing evidence, rather than predicting play itself (Gemmell et al., 2022). The theoretical framework underlying the metric aligns closely with findings from other evidence reviews on neighborhood characteristics supportive of children’s outdoor play (Lambert et al., 2019; E.-Y. Lee et al., 2021; H. Lee et al., 2015; Visser & van Aalst, 2022). The metric was developed using indicators corresponding to five themes identified in the theoretical framework, with indicator selection and weighting informed by a survey of experts in the fields of outdoor play, urban planning, child health and development, child physical activity, injury prevention, child’s rights, and environmental health (Gemmell et al., 2026). The lower age range of two years represents when children may begin to semi-independently experience the outdoor environment and the upper age range of six years represents when children may undergo a transition in routines and environments as they enter primary school. The playability metric utilizes multiple open data sources georeferenced to residential six-digit postal codes across the 35 largest census metropolitan areas in Canada.

The playability metric integrates neighborhood features identified as influencing the availability, accessibility, and acceptability of the space for outdoor free play and operationalizes them into five domains: (1) traffic environment, which captures traffic and pedestrian settings that influence access to and spaces for outdoor free play (indicators: road types, intersections, walking routes, cycling routes); (2) spaces for play, which captures the availability of formal and informal spaces that are designed for or may be adapted for play (indicators: parks, playgrounds, open spaces not part of a building footprint); (3) social environment, which captures the presence of other children and factors that may influence social connection to local people, places and institutions and sense of social safety (indicators: residential mobility, recent immigration, children under 14 years); (4) natural environment, which captures the presence of and opportunity to interact with nature (indicators: greenness, tree canopy, water); and (5) child-relevant destinations, which captures local destinations that may motivate active travel and incidental play and foster local social connections and knowledge of local communities (indicators: schools, childcare centres, library and community centres, recreational facilities). The theoretical validity of the domains and indicators is supported by detailed documentation of evidence informing their selection (see Supplementary Tables A1-A7 of Gemmell et al., 2026). For instance, prior quantitative and qualitative evidence have demonstrated strong conceptual links between parental perceptions of neighborhood traffic safety, including traffic volume, speed, and safe walking and cycling routes, and young children’s outdoor free play (Abu-Ghazzeh, 1998; Andrews et al., 2019; Bassul et al., 2021; Dwyer et al., 2008; Hunter et al., 2022). In addition, intersections have been negatively associated with young children’s outdoor play (Aarts et al., 2012). Based on this evidence, indicators of neighborhood traffic exposure, walking and cycling routes, and intersections were developed using available geospatial data, considering buffer sizes most relevant to young children’s outdoor free play (Gemmell et al., 2026). Domain indicators were selected and weighted with consideration of results from the expert survey. For each domain, additive aggregation was used to assign a domain score calculated as the weighted mean of all indicators within the domain. A composite playability score was calculated as the sum of domain scores weighted according to the expert survey results. The composite and domain playability scores were scaled to a range of 0.0001 (lowest) to 10 (highest), with higher scores corresponding to greater playability.

We used the composite playability score and all five domain scores as indicators of neighborhood playability. We assigned playability scores to children using six-digit residential postal codes recorded in the healthcare system that were summarised on an annual basis by Population Data BC. In circumstances where multiple unique postal codes were reported for a child in a single year, Population Data BC selected the latest recorded postal code for the year. The six-digit postal code is a common geographic identifier applied in Canadian environmental epidemiological studies and generally corresponds to a block face or single multi-unit building in urban areas, with a median positional accuracy within 110 to 160 m for urban residential houses and apartments (Khan et al., 2018). The average number of households within a six-digit postal cover is approximately 19, but the number can vary greatly (Statistics Canada, 2009). In general, the spatial extent and population size of six-digit postal codes is smaller in urban compared to rural areas (El Emam et al., 2011). For the composite playability score and each of the five domain scores, we calculated a time-weighted early-life exposure estimate as the mean of annual playability scores across the exposure period (from age two to time of EDI assessment), while accounting for residential changes reported in the context of the healthcare system.

### 2.4 Potential confounders

We included potential confounders of the association between neighbourhood playability and early childhood development chosen based on theoretical and empirical grounds, in addition to data availability. The variables included in the model are shown in the directed acyclic graph (DAG) (Textor et al., 2011) in Figure S1. We included individual-level variables for child’s age at EDI assessment, sex (female, male), and English as a second language (no, yes). While not hypothesized to be related to neighborhood playability, child’s age and sex are routinely controlled for in prior studies and have been found to be associated with EDI scores (Guhn et al., 2010, 2016; Thomson et al., 2017). We used English as a second language as a proxy for a child’s cultural and/or ethnic background, which may reflect diverse linguistic and cultural contexts that can shape developmental trajectories and may influence selection of residential location (Guhn et al., 2016; Milbrath & Guhn, 2019). Information on individual-level socio-economic status was not available, so we selected data on maternal age (<25, 25-40, >40) and lone parent household status (no, yes) at time of birth as proxies of household socio-economic status. Lower maternal age at time of birth and belonging to a lone parent household have been associated with lower household socio-economic status in childhood, while higher maternal age has been associated with higher childhood household socio-economic status (Adams & Ryan, 2000; van Roode et al., 2017). In addition, we used health practitioner Premium Subsidy Codes recorded in the MSP, that indicate the rate of provincial health insurance premium subsidy, as a proxy for low household income. In line with prior research (Guhn et al., 2020), we created a binary variable (no, yes) to indicate whether a child received subsidized MSP for the earliest recorded health practitioner visit. Eligibility for opting-in for MSP subsidy is based on a household’s adjusted net income and subsidy rate thresholds are adjusted over time to reflect changes in standard of living (e.g., the cut-off for subsidy incrementally increased from $20,000 in 2000 to $30,000 in 2010). Socio-economic status has been associated with neighborhood environmental exposures and childhood developmental outcomes (Doiron et al., 2020; Gill et al., 2024; Guhn et al., 2020; Janus & Duku, 2007).

We assessed neighborhood-level socio-economic status using the material deprivation and residential instability dimensions of the 2006 Canadian Marginalization (CAN-Marg) Index (Matheson et al., 2012). The material deprivation dimension indicates education, employment, income, and family structure measures, while the residential instability dimension indicates housing type, housing ownership, residential moves during the past five years, and household members’ demographic and relationship characteristics. The CAN-Marg summarizes each dimension within dissemination areas, which are small geographic units comprised of 400 to 700 individuals (Statistics Canada, 2021). We assigned material deprivation and residential instability values to children using six-digit postal codes (Pinault et al., 2020), with higher values corresponding to areas of greater marginalization. Previous Canadian epidemiological studies have used CAN-Marg and have associated the index with health and behavioral outcomes (Matheson et al., 2012; White et al., 2013).

### 2.5 Statistical analysis

We first calculated descriptive statistics of the analytic sample. We then used multilevel linear regression models to characterize the cross-sectional association between neighborhood playability and early childhood development, with total EDI score as the outcome, neighborhood playability as a fixed effect, and teacher as a random effect to account for teacher-rater bias and classroom clustering. We tested for residual independence with Moran’s *I* statistic and found no evidence of spatial autocorrelation, so we did not include spatial dependence in our statistical models. In each model, we regressed the outcome on a single explanatory variable (composite playability and each playability domain score) to evaluate the association of each exposure with childhood development independently. We modelled composite and domain playability scores as quartiles and evaluated the associations by comparing higher quartiles of neighborhood playability to the lowest quartile of neighborhood playability (Q1; reference). We tested for multicollinearity among the covariates with generalized variance-inflation factors. With the largest generalized variance-inflation factor of 1.12, we found little evidence of multicollinearity and, therefore, we controlled for all eight covariates in the adjusted models. Given that lower early childhood developmental outcomes have been observed among male children and children who experience lower socio-economic status (Gill et al., 2024; Guhn et al., 2020; Talarico et al., 2023), we tested for potential effect modification by a child’s sex and socio-economic status (proxied by MSP subsidy) through stratified analyses and models with interaction terms between the exposure and modifier, adjusting for confounders. We excluded from our analysis children with missing or invalid data for any of the outcome (n = 3,807; 10.1%), exposure (n= 1,087; 2.9%), or confounder (n = 1,507; 4.0%) variables. All analyses were performed in R version 4.0.5 (R Core Team, 2023).

In addition to evaluating the association between neighborhood playability and overall childhood development, as measured by total EDI score, we ran complementary analyses to evaluate the associations between neighborhood playability and the five EDI domain scores (range: 0-10) to assess the consistency of results across dimensions of early childhood development. We also conducted sensitivity analyses to test the robustness of the findings to a different measure of early childhood development. Specifically, we evaluated the association between neighborhood playability and early childhood developmental vulnerability (no, yes) using multi-level logistic regression models. Children were classified as vulnerable in their overall EDI score if any one of their five EDI domain scores were within the bottom 10^th^ percentile of a provincial distribution (Janus & Duku, 2007).

## 3 Results

### 3.1 Characteristics of the study sample

The final study sample included 30,126 children (Figure S2). Descriptive and correlation analyses demonstrated small differences between children who were included and excluded from our analytical sample (Table S1). Specifically, compared to children who were excluded, our sample had a slightly higher proportion of children who were in the English as a second language group and who were born to mothers aged 25 to 40 years and a slightly lower proportion of children from households with lone parents and who received subsidized MSP.

Table 1 summarizes the characteristics of our study sample. Children were on average 5.6 years of age at the time of EDI assessment, 48.4% were females, and 32.1% were in the English as a second language group. Most children were born to mothers aged 25 to 40 years (86.6%) and belonged to households with more than one parent (96.3%). One fifth of children lived in low-income households who received subsidized MSP (20.3%). Total EDI score was negatively skewed, with children receiving a mean total EDI score of 40.2 (out of possible range of 0-50). The mean neighborhood composite playability score was 6 (out of a possible range of 0-10). Within the individual playability domains, the highest mean score was for social environment (6.7) and the lowest mean score was for child-relevant destinations (1.6).

### 3.2 Early childhood development in association with neighborhood playability

Results from the adjusted models of the association between neighborhood playability and early childhood development are presented in Table 2 (see Table S2 for unadjusted results). In adjusted models, children living in neighborhoods with the highest quartile of the composite playability score had higher total EDI scores compared to children living in neighborhoods with the lowest quartile of the composite playability score (b: 0.30, 95% CI: 0.05, 0.54). A post-hoc analysis that z-standardized total EDI score, so that regression coefficients could be interpreted similarly to Cohen’s d (Cohen, 1992; Milbrath & Guhn, 2019), indicated that the effect size of this association was small (Cohen’s *d* = 0.04; Table S3). Positive associations were also found for children living in neighborhoods with the highest quartile of the natural environment (b: 0.78, 95% CI: 0.50, 1.06), social environment (b: 0.34, 95% CI: 0.08, 0.60), and traffic environment (b: 0.32, 95% CI: 0.07, 0.56) domain scores, while a negative association was found for children living in neighborhoods with the highest quartile of the child-relevant destinations domain score (b: -0.40, 95% CI: -0.64, -0.15); the effect sizes of these associations were small (Table S3). The spaces for play domain score showed a negative association with total EDI score, though the confidence interval range included the null. A post-hoc analysis that included all playability domain scores in a single model yielded similar findings to the main results, though children living in neighborhoods with the highest quartile of the spaces for play domain score had lower total EDI scores compared to those living in neighborhoods with the lowest quartile (Table S4).

Table 3 shows results from models testing for effect modification by a child’s sex and socio-economic status. Overall, there was stronger evidence for effect modification by sex than by socio-economic status, as indicated by statistically significant interaction terms for several domains. Stratified models indicated that positive associations between higher quartiles of the neighborhood composite playability score on total EDI score were generally only present for males and children who did not receive MSP subsidy; however, interaction terms were only statistically significant for child’s sex (p = 0.023), suggesting evidence of sex-based effect modification and weak evidence for socio-economic status. Stratified models indicated positive associations of the natural environment domain score on total EDI score were generally present across groups but were stronger for males and children who received MSP subsidy. Interaction terms were only statistically significant for child’s sex (p = 0.017), indicating evidence of sex-based effect modification and weak evidence for socio-economic status. Stratified models and interaction terms indicated positive associations between higher quartiles of the social environment domain score and total EDI score were stronger for males. Though the interaction term was significant for MSP subsidy (p = 0.030), the confidence interval ranges crossed the null across groups, indicating weak evidence of socio-economic effect modification for this domain. Conversely, stratified models indicated negative associations between the highest quartile of neighborhood spaces for play domain score and total EDI score were stronger for females and children who received MSP subsidy. Interaction terms were only statistically significant for MSP subsidy (p = 0.0050), indicating evidence of effect modification by socio-economic status and weak evidence for sex. While differences in associations were observed in stratified models, interaction terms were not statistically significant (p ranging from 0.10 to 0.42) for the neighborhood traffic environment and child-relevant destinations domain scores, indicating weak evidence of effect modification for these domains.

### 3.3 Sensitivity analyses

Consistent with our main findings, we found that children living in neighborhoods with the highest quartiles of composite playability, traffic environment domain, social environment domain, and natural environment domain scores had higher scores across EDI domain scores compared to children living in neighborhoods with the lowest quartile of these exposures, while lower EDI domain scores were found for children living in neighborhoods with the highest quartile of the child-relevant destinations domain score (Table S5). For each playability score, the direction and relative strength of the estimated effects were similar across EDI domains. When we measured EDI as a binary indicator in the sensitivity analyses, we found that children living in neighborhoods with the highest quartile of playability, including domain scores for social environment and natural environment, had lower odds of being vulnerable to poor childhood development as compared to children living in neighborhoods with the lowest quartile of these exposures (adjusted OR: 0.90; 95% CI: 0.82, 0.98 for composite playability; Table S6). Conversely, children living in neighborhoods with the highest quartile of child-relevant destinations had higher odds of being vulnerable to poor childhood development.

## 4 Discussion

In this large population-based birth cohort study, we observed a positive association between early-life composite neighborhood playability and early childhood development after adjustment for demographic and socio-economic factors, equivalent to a small effect size. Furthermore, we found that associations between neighborhood playability and EDI scores varied in direction and strength across individual playability domains. Specifically, the strongest positive association with total EDI score was found for the natural environment domain score, followed by the social environment and traffic environment domain scores. Higher neighborhood child-relevant destination domain scores were negatively associated with early childhood development. Our results suggested a negative association between the spaces for play domain score and early childhood development, though the confidence interval range included the null in our main analyses. We observed that positive associations for composite playability and the social and natural environment domain scores were stronger among males, while negative associations for the spaces for play domain score were stronger among children from low-income households.

### 4.1 Early-life neighborhood playability may improve early childhood development

We found that children living in neighborhoods with higher composite playability had higher EDI scores. The observed difference of 0.30 on total EDI score attributable to children living in a neighborhood with the highest compared to the lowest quartile of composite playability was equivalent to a small effect size (Cohen’s *d* = 0.04). Furthermore, results from our sensitivity analysis indicated that children living in neighborhoods with the highest quartile of composite playability, as compared to those in the lowest quartile, had 10% lower odds of being vulnerable to poor childhood development. Though our observed associations were weak, small effects on EDI distributed across an entire population may have substantial benefits on children’s health and development. Higher composite neighborhood playability scores could shift the population distribution so that a higher percentage of children are meeting age-appropriate developmental expectations and, importantly, a lower the percentage of children are experiencing developmental vulnerabilities. Given the lack of known studies exploring the association between neighborhood playability, as measured through a composite index, and early childhood development, our findings contribute new evidence to this research area. Broadly, our findings are consistent with previous research linking neighborhood characteristics (Christian et al., 2015; Gascon et al., 2016; Minh et al., 2017) and outdoor free play (Brussoni et al., 2015; Dankiw et al., 2020; Gibson et al., 2017; Gray et al., 2015) with positive outcomes for childhood health and development.

The small effect sizes observed in our study are consistent with prior research that has indicated that individual demographic and socio-economic factors may be more important in explaining variations in EDI outcomes among children than characteristics of the neighborhood environment (Christian et al., 2017; Turrell et al., 2020; Villanueva et al., 2023). For example, the effect sizes in our study are smaller than those reported by Milbrath and Guhn (2019), who found a strong negative association between poverty and total EDI scores, with a larger effect size for family-level (Cohen’s *d* = -0.54) than neighborhood-level (Cohen’s *d* = -0.14) socioeconomic disadvantage. Furthermore, a study using population-level data in Alberta, Canada found that the main risk factors for developmental vulnerability on the EDI among kindergarteners were a child’s history of mental health diagnosis (risk ratio (RR) = 1.46), male sex (RR = 1.51), and low socio-economic status (RR = 1.58) (Talarico et al., 2023). The odds ratios estimated in our sensitivity analysis are weaker relative to the main risk factors reported by Talarico and colleagues, however, our findings of lower odds of developmental vulnerability for children living in the highest as compared to the lowest quartile of composite playability are similar in magnitude to the risk ratios for breast-feeding status (RR = 0.87) and child’s chronic disease status (RR = 1.13), and are relatively stronger than the risk ratios for higher community education level (RR = 0.99), child’s emergency health service use (RR = 1.01), and not speaking English or French (RR = 1.05) observed in their study.

### 4.2 Associations vary across domains of neighborhood playability

While composite playability was positively associated with early childhood development, we found that associations for individual playability domain scores varied in direction and strength. We found the strongest positive association with early childhood development for the natural environment playability domain, indicating that available green and blue spaces may be particularly important neighborhood playability characteristics for children’s health and development; though, the effect size was small (Cohen’s *d* = 0.10 for the highest quartile). Our finding of a positive association is consistent with earlier studies that found that higher early-life residential exposure to vegetation (Jarvis et al., 2021), particularly tree cover (Jarvis et al., 2022), was associated with higher total EDI scores and lower odds of vulnerability to poor childhood development. Prior research shows that residential green space supports positive childhood health and developmental outcomes (Davis et al., 2021), and blue space use has been associated with parent perceptions of children’s positive development (George et al., 2024) and improved socio-emotional outcomes among children (Amoly et al., 2014). While Dzhambov et al. (2022) reported that associations between green space and blood pressure among 8- to 12-year-old children were partially explained by more outdoor play spaces, Mygind et al. (2022) did not observe evidence of mediation of time spent playing on the association between home yard vegetation and socio-emotional function. Research highlights that natural spaces are attractive settings to children and that they afford natural play materials, such as sticks and leaves, and encourage more diverse and creative play than other settings (Chawla, 2015; Gemmell et al., 2022), which may in part explain the stronger positive association with early childhood development that we observed for the natural environment domain relative to the other playability domains.

We also observed positive early childhood development outcomes among children living in neighborhoods with higher social environment and traffic environment playability domain scores; though, effect sizes for the highest quartile of these domains (Cohen’s *d* = 0.04) were less than half the effect size observed for the natural environment domain. Our finding of a positive association for the social environment playability domain is consistent with research linking greater parent-perceived neighborhood social cohesion and collective efficacy to improved childhood behavior (Hosokawa & Katsura, 2020) and physical and mental health in adolescence (Kronaizl & Koss, 2023), with the later finding partially explained by more time spent in play during middle childhood. Conversely, low levels of neighborhood social cohesion have been associated with poor childhood development, including lower verbal ability scores and higher behavioral problems (Caughy et al., 2008; Kohen et al., 2002).

Our finding of a positive association between the traffic environment domain score and total EDI score aligns with Christian et al. (2017), who observed that neighborhoods with fewer main roads had a lower proportion of children vulnerable on the social domain of the AEDC, and Bell et al. (2020) who reported that higher neighborhood road traffic exposure was associated with higher odds of social and emotional vulnerability among Australian children. High traffic environments may act as a barrier for children’s accessibility to play spaces (Tavakoli et al., 2024) and be associated with higher levels of traffic-related air pollution and noise levels, all of which may adversely affect children’s health and development (Castagna et al., 2022; Liu et al., 2024; Zare Sakhvidi et al., 2018). Conversely, the presence of child-friendly walking and cycling routes enables safe access to local destinations and facilitates incidental play, physical, and social activities along routes (Gemmell et al., 2022), which can support improved childhood development (Hosokawa & Katsura, 2020; Ortegon-Sanchez et al., 2025; Robinson et al., 2022).

Contrary to our initial hypothesis, we found a negative association between the child-relevant destinations domain score and early childhood development. Prior research in this area is mixed, with some studies reporting positive (Ghandour et al., 2021; Lipscomb et al., 2019; Prado-Galbarro et al., 2021; Turrell et al., 2020), negative (Christian et al., 2017), and mixed (Collyer et al., 2022; Villanueva et al., 2023) associations between the number and proximity of child-relevant destinations and childhood developmental outcomes. These inconsistencies suggest that the mere presence of child-relevant destinations may be less important for supporting childhood development than the quality of the space and services provided. The playability metric defines child-relevant destinations based on public rather than private programming and the domain score has been negatively correlated with area-level material advantage (Gemmell et al., 2026), suggesting that the destinations and services captured by the metric may lack the-resources to effectively support early childhood development. In addition, Gemmell et al. (2026) found that the child-relevant destination domain score was positively correlated with population density, suggesting that neighborhoods with more child-relevant destinations may be more densely populated. They also found that the natural environment domain score was negatively associated with both population density and the child-relevant destinations domain score (Gemmell et al., 2026). This pattern suggests that higher scores on the child-relevant destination domain may partly reflect underlying physical features of the urban form, including denser development, reduced natural environments, greater impervious surface coverage, and differences in housing and building types (e.g., multi-unit housing) (Dempsey et al., 2010; Jarvis et al., 2020). Although findings on density and child health are mixed (Dau et al., 2025), some studies have found that higher population density is inversely associated with childhood health and developmental outcomes (Nordbø et al., 2020; Prado-Galbarro et al., 2021), perhaps due to higher exposures to traffic noise and crime (Binter et al., 2022). Higher population density may also place greater strain on local services, creating barriers to accessing and benefitting from child-relevant destinations. Together these factors may help explain the observed negative association between child-relevant destinations and early childhood development. It is possible that the observed negative association between the availability of child-relevant destinations and early childhood development may also reflect reduced independent mobility among young children in Metro Vancouver; thus, despite availability, children may not travel to, use, and benefit from these spaces.

Finally, we found a potential negative association between the spaces for play domain score and early childhood development. This negative trend may be explained by the domain measurement, which captures the availability of both formal (i.e., parks, playgrounds, recreation areas) and informal (i.e., open space not covered by building footprint) spaces. While formal play spaces have been positively associated with children’s health and development (Acolin et al., 2022; Alderton et al., 2022; Feng & Astell-Burt, 2017a, 2017b; Pérez-del-Pulgar et al., 2021; Putra et al., 2021; Turrell et al., 2020), findings for informal spaces are mixed. For instance, Christian et al. (2017) found that larger quantities of home yard space lowered odds of emotional vulnerability, while Bell et al. (2020) found that home yard area increased odds of physical and social vulnerability. Informal spaces may include impervious surfaces (e.g., roads, parking lots), which have been associated with lower total EDI scores and higher odds of vulnerability to poor childhood development (Jarvis et al., 2022), potentially due to increased noise, air pollution, and heat exposure. The potentially higher levels of environmental stressors within informal play spaces may offset the benefits of formal play spaces, possibly explaining the negative association observed in our study.

### 4.3 Associations may vary by child’s sex and household socio-economic status

We observed that some associations differed according to a child’s sex and household socio-economic status. Results from stratified models and models with interaction terms indicated that positive associations for neighborhood composite playability and the social and natural environment domain scores with early childhood development were stronger among males compared to females. While inconsistencies exist, a recent review found that boys generally engage in more outdoor free play compared to girls (Gemmell et al., 2022), and that boys may be more likely to play with natural elements (Sargisson & McLean, 2012). Some prior studies have reported more consistent and stronger health associations of green space among males (Markevych et al., 2014; Putra et al., 2021), while others have observed null (M. Lee et al., 2019) or mixed (Richardson et al., 2017) findings for effect modification by sex. Differences may in part be due to higher levels of parental-perceived safety of outdoor free play for male compared to female children and gendered social norms influencing engagement in outdoor free play (Remmers et al., 2014; Son & Kim, 2024).

Next, we observed that negative associations between the spaces for play domain score and early childhood development were stronger among children with lower household socio-economic status (i.e., did receive MSP subsidy). Some studies have observed stronger positive associations between spaces for play, such as open spaces and parks, and childhood development among children from higher socio-economic groups (Alderton et al., 2022; Collyer et al., 2022) and others among lower socio-economic groups (Pérez-del-Pulgar et al., 2021). The stronger negative association for children with lower household socio-economic status observed in our study may reflect lower parental resources, time, and support for outdoor free play. Previous studies have shown that financial stress, long work hours, and parental focus on making a living may negatively impact young children’s engagement in outdoor free play as parents must focus on financial survival (Penilla et al., 2017). It may also reflect that children reside in lower socio-economic neighborhoods and experience more restrictions to outdoor free play related to the availability and quality of environmental characteristics supportive of play (Parent et al., 2021).

### 4.4 Strengths and limitations

Our study has several strengths. We addressed important gaps in the literature by being the first known study to examine the association between neighborhood playability and early childhood development. Using a novel playability metric, we were able to assess the associations of overall composite neighborhood playability, in addition to estimating and comparing the associations of individual playability domains. This methodology provided important nuanced information on associations between the neighborhood environment and children’s early development. While our study was limited to Metro Vancouver, Canada, the playability metric was developed using international literature and expert input, facilitating the generalization of our exposure measurement framework to diverse global settings. In addition, we used a population-based study design, which reduces potential sources of sample bias by permitting a more representative sample of our study region. Finally, the validity and reliability of our teacher-rated EDI outcome measure has been well-established in prior studies (Forget-Dubois et al., 2007; Janus et al., 2011; Janus & Offord, 2007).

We recognise several limitations of our study. The playability index is a theoretically valid measure of the supportiveness of the environment for outdoor free play, but it is not a measure of play itself and we did not have data on play behavior for children in our study cohort. Future research with empirical data is needed to confirm the validity and reliability of the neighborhood playability metric, as these properties remain to be tested for this newly developed metric. While research confirms neighborhood characteristics captured by the playability metric are associated with more outdoor free play and that outdoor play is associated with improved childhood development, future studies should formally test outdoor free play as a mediating factor linking neighborhood playability to early childhood development. In addition, we cannot infer causality due to the lack of longitudinal data on the EDI outcome; though, we were able to demonstrate a temporal sequence in the association because residential exposures preceded the outcome measure. It is possible that some portion of the observed association between early-life neighborhood playability and early childhood development could have resulted from residential self-selection of families with more resources and healthier lifestyles into neighborhoods more supportive of outdoor free play. When possible, future studies should adopt longitudinal study designs to analyse changes over time to better determine if a causal relationship exists. Next, since the administrative data we used were not collected for research purposes, we lacked some individual-level variables that may have led to residual confounding. To address this limitation, we used all available variables to operationalize factors for which we did not have direct measures (e.g., MSP subsidy as a proxy for low household income). In addition, exposure misclassification may have arisen from the temporal misalignment of the playability metric (data sources ranging from 2010 to 2023) (Gemmell et al., 2026) and our study period (2000 to 2011), in addition to the spatial misalignment between a children’s residential postal code and their exact residential address; though, the spatial error is expected to be minimal due to the small geographic area covered by urban postal codes (Khan et al., 2018). We acknowledge that assigning children’s exposure using residential postal codes may not reflect the settings where children and their families spend time and where health effects are most likely to occur, consistent with the uncertain geographic context problem (Kwan, 2012). Moreover, our exposure measure did not account for the qualitative attributes (e.g., public vs private access, presence of amenities) and use of the surrounding neighborhood environment, which may be important for outdoor free play behaviors and subsequent benefits to early childhood development. Furthermore, we may have excluded some children from our cohort as the BC Perinatal Data Registry does not include data on all pregnancies in BC. The Perinatal Data Registry does not include data on deliveries that are therapeutically or spontaneously delivered at <20 weeks’ gestation and are <500 grams fetal birth weight nor does it include data for unattended deliveries or deliveries at home attended by an unregistered healthcare provider (Perinatal Services BC, 2025). Finally, our study sample was limited to children residing in Metro Vancouver and largely comprised of children who were born to multi-parent households and mothers aged 25-40, who belonged to households that did not receive MSP subsidy, and who spoke English as their first language. As such, our findings may not be generalizable to other regional contexts or populations.

## 5 Conclusions

In this population-based birth cohort study, we identified several small but positive associations between early-life neighborhood playability and early childhood development. Our findings contribute to improved understanding of the influence of the surrounding neighborhood environment on children’s development by assessing associations for both composite and individual domains of playability. Our results suggest that natural environments, including green and blue spaces, may be particularly important for supporting early childhood development. While the size of estimated associations between measures of neighborhood playability and continuous EDI scores were small, our sensitivity analyses highlighted lower odds of being vulnerable to poor childhood development among children residing in neighborhoods with higher levels of playability. The potential of neighborhood playability to shift population-level childhood developmental outcomes upwards and reduce the risk of poor childhood development are important public health benefits. While our study provides a valuable starting point, further research is needed to confirm our findings of a positive association between neighborhood playability, including individual playability domains, and children’s early development and to further refine hypotheses linking these environmental features to children’s experiences and subsequent developmental outcomes. While we did not have data on children’s engagement with play, future research should formally test play as a mediating pathway linking neighborhood playability and early childhood development. If confirmed by future studies, our findings can inform planning and policy practitioners on the design of neighborhood environments for supporting children’s engagement in health-promoting behaviors, like outdoor free play, and improved early childhood development.

## Supplementary Material

Strobe Checklist

Supplementary Material

## Figures and Tables

**Figure 1:**
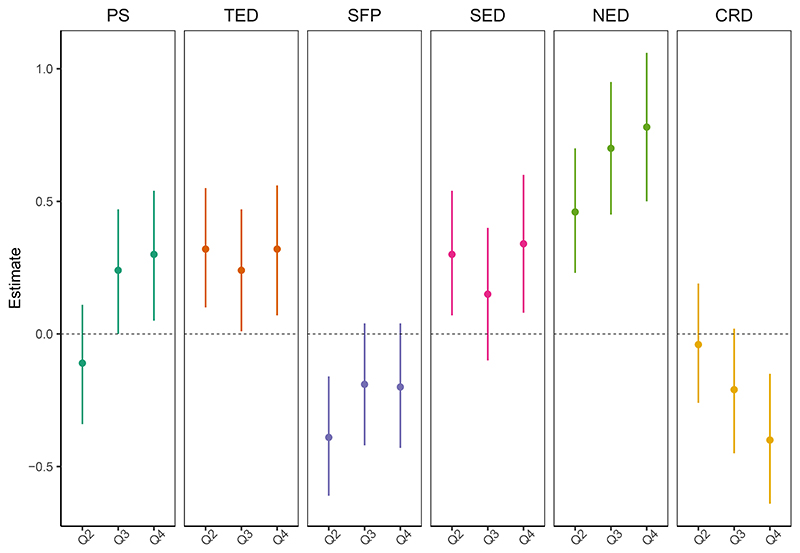
Adjusted associations between neighborhood playability and early childhood development among Metro Vancouver children (n= 30,126) as indicated in Table 2. PS, composite playability score; TED, traffic environment domain score; SFP, spaces for play domain score; SED, social environment domain score; NED, natural environment domain score; CRD, child-relevant destinations domain score

**Table 1:** Characteristics of cohort of children to examine the association between neighborhood playability and early childhood development in Metro Vancouver.

	Total sample (n=30,126)^1^
**Demographic variables**	
Age at EDI assessment	5.6 (0.3)
Sex	
Female	14,581 (48.4%)
Male	15,545 (51.6%)
English as second language	
No	20,451 (67.9%)
Yes	9,675 (32.1%)
MSP subsidy	
No	23,998 (79.7%)
Yes	6,128 (20.3%)
Maternal age	
<25	3,228 (10.7%)
25-40	26,094 (86.6%)
>40	804 (2.7%)
Lone parent household	
No	29,011 (96.3%)
Yes	1,115 (3.7%)
Neighborhood-level material deprivation	-0.3 (0.7)
Neighborhood-level residential instability	-0.1 (0.9)
**Outcome variable**	
Total EDI score	40.2 (7.8)
**Environmental exposure variables**	
Composite playability score	6.0 (0.9)
Traffic environment domain score	6.4 (1.0)
Spaces for play domain score	4.0 (0.8)
Social environment domain score	6.7 (1.0)
Natural environment domain score	4.5 (1.1)
Child-relevant destinations domain score	1.6 (1.2)

1 n (%); Mean (SD)

EDI, Early Development Instrument; MSP, Medical Services Plan.

All values are rounded.

**Table 2:** Adjusted associations between neighborhood playability and early childhood development among Metro Vancouver children (n= 30,126).

Exposure	Total EDI score b-coefficient (95% CI)
Composite playability score	
Quartile 2	-0.11 (-0.34, 0.11)
Quartile 3	0.24 (0.00, 0.47)
Quartile 4	0.30 (0.05, 0.54)
Traffic environment domain score	
Quartile 2	0.32 (0.10, 0.55)
Quartile 3	0.24 (0.01, 0.47)
Quartile 4	0.32 (0.07, 0.56)
Spaces for play domain score	
Quartile 2	-0.39 (-0.61, -0.16)
Quartile 3	-0.19 (-0.42, 0.04)
Quartile 4	-0.20 (-0.43, 0.04)
Social environment domain score	
Quartile 2	0.30 (0.07, 0.54)
Quartile 3	0.15 (-0.10, 0.40)
Quartile 4	0.34 (0.08, 0.60)
Natural environment domain score	
Quartile 2	0.46 (0.23, 0.70)
Quartile 3	0.70 (0.45, 0.95)
Quartile 4	0.78 (0.50, 1.06)
Child-relevant destinations domain score	
Quartile 2	-0.04 (-0.26, 0.19)
Quartile 3	-0.21 (-0.45, 0.02)
Quartile 4	-0.40 (-0.64, -0.15)

Data presented are b-coefficient (95% confidence interval) for each quartile of exposure (reference = quartile 1). Models control for age at time of EDI assessment, sex, English as a second language, lone parent household, maternal age, Medical Services Plan (MSP) subsidy, neighborhood-level material deprivation, and neighborhood-level residential instability. Models include random effect for teacher ID.

All estimated effects and 95% confidence intervals are rounded.

EDI, Early Development Instrument.

**Table 3:** Adjusted associations from stratified and interaction term models testing for effect modification by child’s sex and socio-economic status (proxied by MSP subsidy).

Exposure	Sex			MSP subsidy		
	Stratified model^1^		Interaction model^2^	Stratified model^1^		Interaction model^2^
	Female (n=14,581)	Male (15,545)	p-value^3^	No (n=23,998)	Yes (n=6,128)	p-value^3^
PS			0.023			0.073
Quartile 2	-0.37 (-0.66, -0.08)	0.12 (-0.23, 0.47)		-0.14 (-0.39, 0.12)	-0.16 (-0.69, 0.37)	
Quartile 3	-0.02 (-0.32, 0.28)	0.48 (0.12, 0.84)		0.24 (-0.01, 0.50)	0.14 (-0.41, 0.70)	
Quartile 4	0.09 (-0.22, 0.40)	0.48 (0.11, 0.85)		0.37 (0.10, 0.63)	-0.22 (-0.81. 0.38)	
TED			0.25			0.10
Quartile 2	0.19 (-0.10, 0.48)	0.45 (0.11 0.80)		0.23 (-0.01, 0.47)	0.66 (0.10, 1.22)	
Quartile 3	0.07 (-0.22, 0.37)	0.44 (0.09, 0.79)		0.18 (-0.07, 0.43)	0.51 (-0.06, 1.07)	
Quartile 4	0.05 (-0.26, 0.36)	0.58 (0.21, 0.95)		0.35 (0.08, 0.61)	0.05 (-0.55, 0.66)	
SFP			0.36			0.0050
Quartile 2	-0.48 (-0.77, -0.19)	-0.39 (-0.73, -0.04)		-0.37 (-0.61, -0.12)	-0.51 (-1.05, 0.03)	
Quartile 3	-0.36 (-0.65, -0.07)	-0.07 (-0.42, 0.27)		-0.20 (-0.45, 0.05)	-0.22 (-0.77, 0.33)	
Quartile 4	-0.38 (-0.68, -0.08)	-0.09 (-0.45, 0.27)		-0.06 (-0.32, 0.19)	-0.87 (-1.45, -0.28)	
SED			0.037			0.030
Quartile 2	-0.03 (-0.33, 0.28)	0.58 (0.22, 0.94)		0.09 (-0.17, 0.35)	0.96 (0.41, 1.51)	
Quartile 3	-0.13 (-0.45, 0.19)	0.32 (-0.05, 0.70)		-0.01 (-0.28, 0.26)	0.40 (-0.20, 0.99)	
Quartile 4	0.21 (-0.12, 0.54)	0.45 (0.05, 0.85)		0.20 (-0.09, 0.49)	0.61 (-0.03, 1.25)	
NED			0.017			0.31
Quartile 2	0.19 (-0.11, 0.49)	0.70 (0.35, 1.06)		0.46 (0.20, 0.72)	0.53 (0.01, 1.05)	
Quartile 3	0.33 (0.01, 0.65)	0.97 (0.59, 1.35)		0.73 (0.45, 1.01)	0.50 (-0.08, 1.08)	
Quartile 4	0.56 (0.21, 0.90)	0.98 (0.56, 1.39)		0.74 (0.44, 1.04)	1.16 (0.46, 1.86)	
CRD			0.42			0.12
Quartile 2	-0.07 (-0.36, 0.22)	0.09 (-0.26, 0.43)		-0.05 (-0.29, 0.19)	0.10 (-0.52, 0.72)	
Quartile 3	-0.11 (-0.41, 0.19)	-0.25 (-0.60, 0.11)		-0.21 (-0.46, 0.04)	-0.13 (-0.74, 0.47)	
Quartile 4	-0.50 (-0.81, -0.19)	-0.23 (-0.60, 0.14)		-0.29 (-0.56, -0.03)	-0.75 (-1.36, -0.13)	

Data presented are b-coefficient (95% confidence interval) for each quartile of exposure (reference = quartile 1).

MSP, Medical Services Plan; PS, composite playability score; TED, traffic environment domain score; SFP, spaces for play domain score; SED, social environment domain score; NED, natural environment domain score; CRD, child-relevant destinations domain score.

1 Stratified models control for age at time of EDI assessment, sex, English as a second language, lone parent household, maternal age, MSP subsidy, neighborhood-level material deprivation, and neighborhood-level residential instability. Models include random effect for teacher ID. Models do not adjust for the modifying variable.

2 Interaction term models include an interaction between exposure and modifier, and control for age at time of EDI assessment, sex, English as a second language, lone parent household, maternal age, MSP subsidy, neighborhood-level material deprivation, and neighborhood-level residential instability. Models include random effect for teacher ID.

3 Indicates the p-value for the interaction term between exposure and modifier that was assessed by a Wald test.
