# Supplementary material for: Neighborhood playability and early childhood development: a population-based birth cohort study
Source: Environ Res. Author manuscript; Available in PMC 2026 Mar 19. (PMC7618905; doi:10.1016/j.envres.2026.124124)
Supplement: Strobe Checklist [file EMS212812-supplement-Strobe_Checklist.docx]

STROBE Statement—checklist of items that should be included in reports of cohort studies

|  | Item No. | Recommendation | Relevant text from manuscript |
| --- | --- | --- | --- |
| **Title and abstract** | 1 | (*a*) Indicate the study’s design with a commonly used term in the title or the abstract | In title: “Neighborhood playability and early childhood development: a population-based birth cohort study”  In abstract: “Using a population-based child cohort (n=30,126) in Metro Vancouver, Canada…” |
|  |  | (*b*) Provide in the abstract an informative and balanced summary of what was done and what was found | In the Methods and Results section of Abstract. |
| Introduction | |  |  |
| Background/rationale | 2 | Explain the scientific background and rationale for the investigation being reported | In paragraphs 1-3, Introduction. |
| Objectives | 3 | State specific objectives, including any prespecified hypotheses | In paragraph 4, Introduction |
| Methods | |  |  |
| Study design | 4 | Present key elements of study design early in the paper | In section 2.1 ‘Study population’, Methods |
| Setting | 5 | Describe the setting, locations, and relevant dates, including periods of recruitment, exposure, follow-up, and data collection | In section 2.1 ‘Study population’, Methods  In section 2.2 ‘Assessment of early childhood development’, Methods |
| Participants | 6 | (*a*) Give the eligibility criteria, and the sources and methods of selection of participants. Describe methods of follow-up | In paragraph 2 of section 2.1 ‘Study population’, Methods  An overview is presented in the flowchart as supplement (Figure S2) |
|  |  | (*b*) For matched studies, give matching criteria and number of exposed and unexposed | - |
| Variables | 7 | Clearly define all outcomes, exposures, predictors, potential confounders, and effect modifiers. Give diagnostic criteria, if applicable | In section 2.2 ‘Assessment of early childhood development’, Methods.  In section 2.4 ‘Potential confounders’, Methods.  In section 2.2 ‘Neighborhood playability’, Methods. |
| Data sources/ measurement | 8* | For each variable of interest, give sources of data and details of methods of assessment (measurement). Describe comparability of assessment methods if there is more than one group | In section 2.2 ‘Assessment of early childhood development’, Methods.  In section 2.4 ‘Potential confounders’, Methods.  In section 2.2 ‘Neighborhood playability’, Methods. |
| Bias | 9 | Describe any efforts to address potential sources of bias | In paragraph 1 of section 2.5 ‘Statistical analysis’, Methods  In paragraph 1 of section 3.1 ‘Characteristics of the study sample’, Results.  Comparison of included and excluded participants is presented as supplement (Table S1).  In section 4.4 ‘Strengths and Limitations’, Discussion |
| Study size | 10 | Explain how the study size was arrived at | In paragraph 2 of section 2.1 ‘Study population’, Methods  In paragraph 1 in section 2.5 ‘Statistical analysis’, Methods  An overview is presented in the flowchart as supplement (Figure S2) |
| Quantitative variables | 11 | Explain how quantitative variables were handled in the analyses. If applicable, describe which groupings were chosen and why | In section 2.4 ‘Potential confounders’, Methods  In section 2.5 ‘Statistical analysis’, Methods |
| Statistical methods | 12 | (*a*) Describe all statistical methods, including those used to control for confounding | In paragraph 1 of section 2.5 ‘Statistical analysis’, Methods |
|  |  | (*b*) Describe any methods used to examine subgroups and interactions | In paragraph 1 of section 2.5 ‘Statistical analysis’, Methods: “…we tested for potential effect modification by a child’s sex and socio-economic status (proxied by MSP subsidy) through stratified analyses and models with interaction terms between the exposure and modifier, adjusting for confounders” |
|  |  | (*c*) Explain how missing data were addressed | In paragraph 1 of section 2.5 ‘Statistical analysis’, Methods: “We excluded from our analysis children with missing or invalid data…” |
|  |  | (*d*) If applicable, explain how loss to follow-up was addressed | In paragraph 2 of section 2.1 ‘Study population’, Methods  An overview is presented in the flowchart as supplement (Figure S2) |
|  |  | (*e*) Describe any sensitivity analyses | In paragraph 2 of section 2.5 ‘Statistical analysis’, Methods |
| **Results** |  |  |  |
| Participants | 13* | (a) Report numbers of individuals at each stage of study—eg numbers potentially eligible, examined for eligibility, confirmed eligible, included in the study, completing follow-up, and analysed | In paragraph 1 of section 2.5 ‘Statistical analysis’, Methods  In paragraph 1 of section 3.1 ‘Characteristics of the study sample’, Results.  An overview is presented in the flowchart as supplement (Figure S2) |
|  |  | (b) Give reasons for non-participation at each stage | An overview is presented in the flowchart as supplement (Figure S2) |
|  |  | (c) Consider use of a flow diagram | Supplement (Figure S2) |
| Descriptive data | 14* | (a) Give characteristics of study participants (eg demographic, clinical, social) and information on exposures and potential confounders | In paragraph 2 of section 3.1 ‘Characteristics of the study sample, Results. |
|  |  | (b) Indicate number of participants with missing data for each variable of interest | In paragraph 1 of section 2.5 ‘Statistical analysis’, Methods.  An overview is presented in the flowchart as supplement (Figure S2; Table S1) |
|  |  | (c) Summarise follow-up time (eg, average and total amount) | In paragraph 2 of section 2.1 ‘Study population’, Methods |
| Outcome data | 15* | Report numbers of outcome events or summary measures over time | In paragraph 2 of section 3.1 ‘Characteristics of the study sample, Results. |
| Main results | 16 | (*a*) Give unadjusted estimates and, if applicable, confounder-adjusted estimates and their precision (eg, 95% confidence interval). Make clear which confounders were adjusted for and why they were included | In section 2.4 ‘Potential confounders, Methods  In paragraph 1 of section 2.5 ‘Statistical analysis’, Methods.  In paragraph 1 of section 3.2 ‘Early childhood development in association with neighborhood playability’, Results |
|  |  | (*b*) Report category boundaries when continuous variables were categorized | In paragraph 1 of section 2.4 ‘Potential confounders’, Methods  In paragraphs 1-2 of section 2.5 ‘Statistical analysis’, Methods |
|  |  | (*c*) If relevant, consider translating estimates of relative risk into absolute risk for a meaningful time period | - |
| Other analyses | 17 | Report other analyses done—eg analyses of subgroups and interactions, and sensitivity analyses | In paragraph 2 of section 3.2 ‘Early childhood development in association with neighborhood playability’, Results  In section 3.3 ‘Sensitivity analyses’, Results |
| **Discussion** |  |  |  |
| Key results | 18 | Summarise key results with reference to study objectives | In paragraph 1, Discussion |
| Limitations | 19 | Discuss limitations of the study, taking into account sources of potential bias or imprecision. Discuss both direction and magnitude of any potential bias | In paragraph 2 of section 4.4 ‘Strengths and limitations’, Discussion |
| Interpretation | 20 | Give a cautious overall interpretation of results considering objectives, limitations, multiplicity of analyses, results from similar studies, and other relevant evidence | In paragraph 1, Discussion.  In section 4.1 ‘Early-life neighborhood playability may improve early childhood development’, Discussion.  In section 4.2 ‘Associations vary across domains of neighborhood playability’, Discussion.  In section 4.3 ‘Associations may vary by child’s sex and household socio-economic status’, Discussion.  In section 4.4 ‘Strengths and limitations’, Discussion  In paragraph 1, Conclusions |
| Generalisability | 21 | Discuss the generalisability (external validity) of the study results | In paragraph 2 of section 4.4 ‘Strengths and limitations’, Discussion |
| **Other information** |  |  |  |
| Funding | 22 | Give the source of funding and the role of the funders for the present study and, if applicable, for the original study on which the present article is based | In paragraph 1 of section ‘Funding’ |

*Give information separately for cases and controls in case-control studies and, if applicable, for exposed and unexposed groups in cohort and cross-sectional studies.

**Note:** An Explanation and Elaboration article discusses each checklist item and gives methodological background and published examples of transparent reporting. The STROBE checklist is best used in conjunction with this article (freely available on the Web sites of PLoS Medicine at http://www.plosmedicine.org/, Annals of Internal Medicine at http://www.annals.org/, and Epidemiology at http://www.epidem.com/). Information on the STROBE Initiative is available at www.strobe-statement.org.
