# Supplementary material for: Neighborhood playability and early childhood development: a population-based birth cohort study
Source: Environ Res. Author manuscript; Available in PMC 2026 Mar 19. (PMC7618905; doi:10.1016/j.envres.2026.124124)
Supplement: Supplementary Material [file EMS212812-supplement-Supplementary_Material.docx]

# Supplementary Material


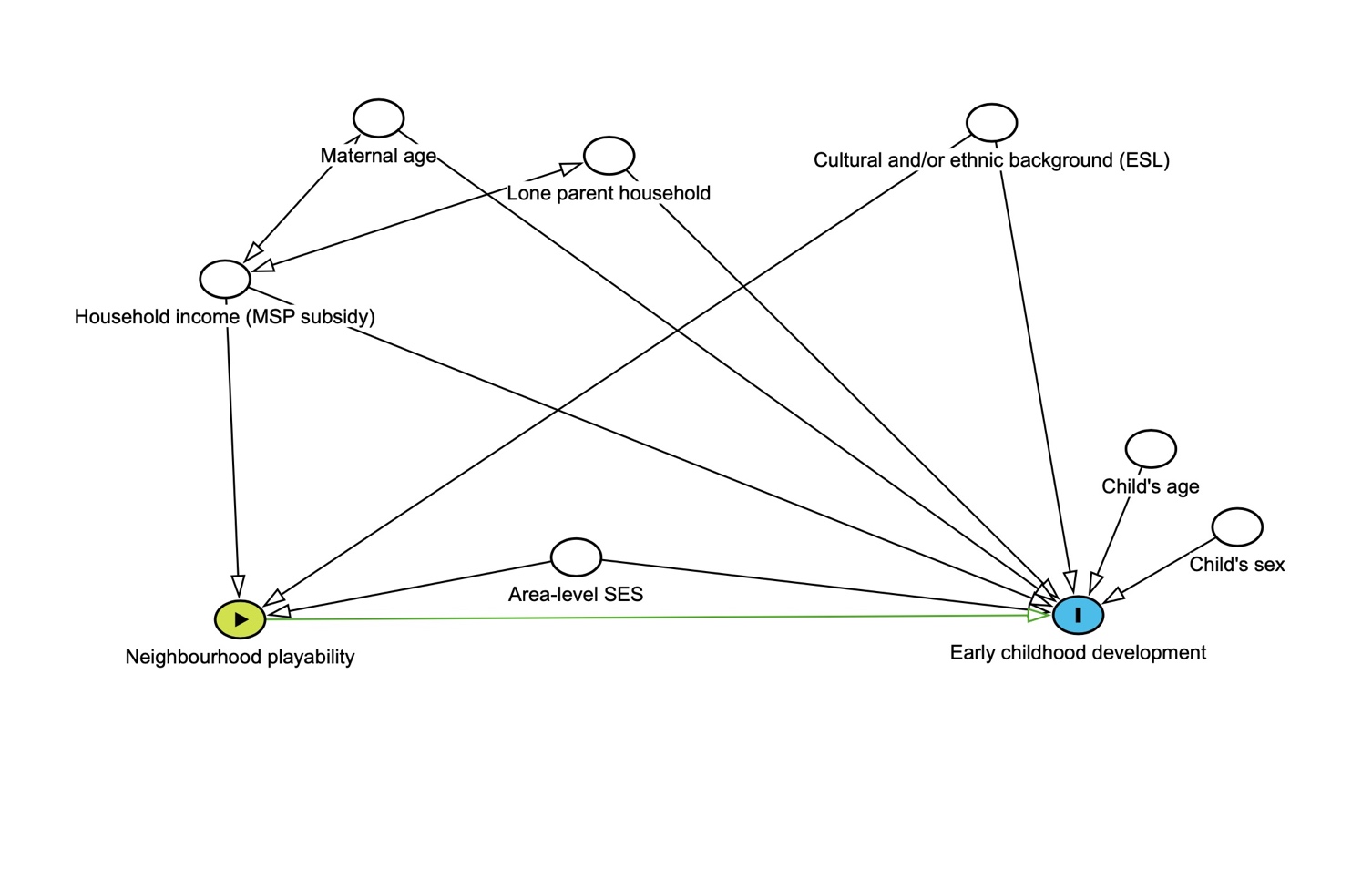


**Figure S1**. Directed acyclic graph (DAG) depicting hypothesized associations between neighborhood playability, potential confounding variables, and early childhood development. DAG created using DAGitty.


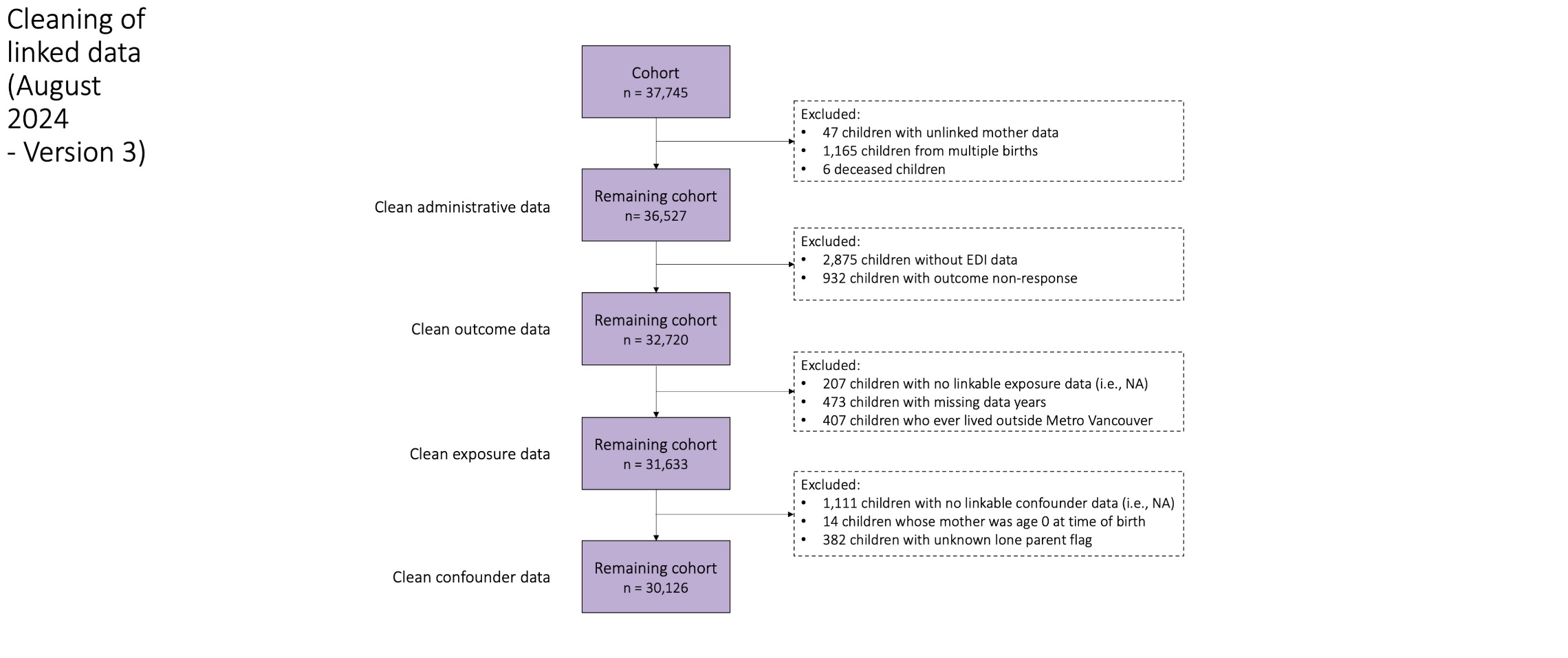


**Figure S2.** Final data cleaning of linked cohort of children to examine the association between neighborhood playability and early childhood development in Metro Vancouver, Canada.

EDI, Early Development Instrument.

**Table S1**. Characteristics of the children who were included and excluded from analytic sample.

|  | *Excluded (n = 6,401)^1^* | *Included (n = 30,126)^1^* | *P-value^2^* |
| --- | --- | --- | --- |
| **Demographic variables** |  |  |  |
| Age at EDI assessment | 5.59 (0.31) | 5.62 (0.29) | <0.001 |
| Missing (n) | 2,875 | 0 |  |
| Sex |  |  | 0.7 |
| Female | 3,059 (48.2%) | 14,581 (48.4%) |  |
| Male | 3,293 (51.8%) | 15,545 (51.6%) |  |
| Missing (n) | 49 | 0 |  |
| English as second language |  |  | <0.001 |
| No | 2,326 (71.5%) | 20,451 (67.9%) |  |
| Yes | 925 (28.5%) | 9,675 (32.1%) |  |
| Missing (n) | 3,150 | 0 |  |
| MSP subsidy |  |  | <0.001 |
| No | 4,496 (74.6%) | 23,998 (79.7%) |  |
| Yes | 1,529 (25.4%) | 6,128 (20.3%) |  |
| Missing (n) | 376 | 0 |  |
| Maternal age |  |  | <0.001 |
| <25 | 1,204 (19.1%) | 3,228 (10.7%) |  |
| 25-40 | 4,969 (78.7%) | 26,094 (86.6%) |  |
| >40 | 141 (2.2%) | 804 (2.7%) |  |
| Missing | 87 | 0 |  |
| Lone parent household |  |  | <0.001 |
| No | 5,147 (84.3%) | 29,011 (96.3%) |  |
| Yes | 474 (7.8%) | 1,115 (3.7%) |  |
| Unknown | 484 (7.9%) | 0 (0.0%) |  |
| Missing | 296 | 0 |  |
| Neighborhood-level material deprivation | -0.25 (0.77) | -0.27 (0.72) | 0.6 |
| Missing | 2,195 | 0 |  |
| Neighborhood-level residential instability | 0.03 (1.00) | -0.09 (0.94) | <0.001 |
| Missing | 2,195 | 0 |  |
| **Outcome variable** |  |  |  |
| Total EDI score | 38.20 (8.72) | 40.19 (7.80) | <0.001 |
| Missing | 3,293 | 0 |  |
| **Environmental exposure variables** |  |  |  |
| Composite playability score | 5.84 (0.93) | 5.97 (0.88) | <0.001 |
| Missing | 3,100 | 0 |  |
| Traffic environment domain score | 6.22 (1.02) | 6.39 (1.02) | <0.001 |
| Missing | 3,100 | 0 |  |
| Spaces for play domain score | 3.94 (0.79) | 3.97 (0.80) | 0.3 |
| Missing | 3,100 | 0 |  |
| Social environment domain score | 6.52 (1.12) | 6.67 (1.04) | <0.001 |
| Missing | 3,100 | 0 |  |
| Natural environment domain score | 4.40 (1.14) | 4.47 (1.07) | <0.001 |
| Missing | 3,100 | 0 |  |
| Child-relevant destinations domain score | 1.62 (1.32) | 1.55 (1.24) | 0.023 |
| Missing | 3,100 | 0 |  |

*^1^* n (%); Mean (SD)

*^2^* Pearson’s Chi-squared test; Wilcoxon rank sum test

EDI, Early Development Instrument; MSP, Medical Services Plan.

Note: There was overlap in exclusion, so missing values for each variable in Table S1 may not correspond perfectly to values in Figure S2.

**Table S2.** Unadjusted and adjusted associations between neighborhood playability and early childhood development among Metro Vancouver children (n= 30,126).

| *Exposure* | *Total EDI score*  *b-coefficient (95% CI)* | |
| --- | --- | --- |
|  | *Unadjusted* | *Adjusted* |
| Composite playability score | | |
| Quartile 2 | 0.25 (0.01, 0.49) | -0.11 (-0.34, 0.11) |
| Quartile 3 | 0.87 (0.62, 1.12) | 0.24 (0.00, 0.47) |
| Quartile 4 | 1.25 (0.99, 1.50) | 0.30 (0.05, 0.54) |
| Traffic environment domain score | | |
| Quartile 2 | 0.24 (0.00, 0.48) | 0.32 (0.10, 0.55) |
| Quartile 3 | 0.08 (-0.17, 0.33) | 0.24 (0.01, 0.47) |
| Quartile 4 | 0.11 (-0.16, 0.37) | 0.32 (0.07, 0.56) |
| Spaces for play domain score | | |
| Quartile 2 | -0.28 (-0.52, -0.04) | -0.39 (-0.61, -0.16) |
| Quartile 3 | 0.03 (-0.22, 0.27) | -0.19 (-0.42, 0.04) |
| Quartile 4 | 0.33 (0.08, 0.59) | -0.20 (-0.43, 0.04) |
| Social environment domain score | | |
| Quartile 2 | 0.59 (0.35, 0.83) | 0.30 (0.07, 0.54) |
| Quartile 3 | 0.76 (0.51, 1.01) | 0.15 (-0.10, 0.40) |
| Quartile 4 | 1.31 (1.05, 1.56) | 0.34 (0.08, 0.60) |
| Natural environment domain score | | |
| Quartile 2 | 1.08 (0.83, 1.33) | 0.46 (0.23, 0.70) |
| Quartile 3 | 1.82 (1.56, 2.07) | 0.70 (0.45, 0.95) |
| Quartile 4 | 2.54 (2.27, 2.81) | 0.78 (0.50, 1.06) |
| Child-relevant destinations domain score | | |
| Quartile 2 | -0.48 (-0.72, -0.24) | -0.04 (-0.26, 0.19) |
| Quartile 3 | -0.89 (-1.14, -0.64) | -0.21 (-0.45, 0.02) |
| Quartile 4 | -1.20 (-1.46, -0.95) | -0.40 (-0.64, -0.15) |

Data presented are b-coefficient (95% confidence interval) for each quartile of exposure (reference = quartile 1).

Adjusted models control for age at time of EDI assessment, sex, English as a second language, lone parent household, maternal age, Medical Services Plan (MSP) subsidy, neighborhood-level material deprivation, and neighborhood-level residential instability. Models include random effect for teacher ID.

All estimated effects and 95% confidence intervals are rounded.

EDI, Early Development Instrument.

**Table S3.** Adjusted associations between neighborhood playability domain scores and early childhood development among Metro Vancouver children, with z-standardized total EDI score (n= 30,126).

| *Exposure* | *Total EDI score^1^*  *β-coefficient (95% CI)* |
| --- | --- |
| Composite playability score | |
| Quartile 2 | -0.01 (-0.04, 0.01) |
| Quartile 3 | 0.03 (0.00, 0.06) |
| Quartile 4 | 0.04 (0.01, 0.07) |
| Traffic environment domain score | |
| Quartile 2 | 0.04 (0.01, 0.07) |
| Quartile 3 | 0.03 (0.00, 0.06) |
| Quartile 4 | 0.04 (0.01, 0.07) |
| Spaces for play domain score | |
| Quartile 2 | -0.05 (-0.08, -0.02) |
| Quartile 3 | -0.02 (-0.05, 0.00) |
| Quartile 4 | -0.03 (-0.06, 0.01) |
| Social environment domain score | |
| Quartile 2 | 0.04 (0.01, 0.07) |
| Quartile 3 | 0.02 (-0.01, 0.05) |
| Quartile 4 | 0.04 (0.01, 0.08) |
| Natural environment domain score | |
| Quartile 2 | 0.06 (0.03, 0.09) |
| Quartile 3 | 0.09 (0.06, 0.12) |
| Quartile 4 | 0.10 (0.06, 0.14) |
| Child-relevant destinations domain score | |
| Quartile 2 | -0.00 (-0.03, 0.02) |
| Quartile 3 | -0.03 (-0.06, 0.00) |
| Quartile 4 | -0.05 (-0.08, -0.02) |

Data presented are β-coefficient (95% confidence interval) for each quartile of exposure (reference = quartile 1).

Adjusted models control for age at time of EDI assessment, sex, English as a second language, lone parent household, maternal age, Medical Services Plan (MSP) subsidy, neighborhood-level material deprivation, and neighborhood-level residential instability. Models include random effect for teacher ID.

All estimated effects and 95% confidence intervals are rounded.

EDI, Early Development Instrument

^1^ Total EDI score was z-standardized, so that β-coefficients can be interpreted as effect sizes like Cohen’s d

**Table S4.** Adjusted associations between neighborhood playability domain scores and early childhood development among Metro Vancouver children, while including all playability domain scores in a single model (n= 30,126).

| *Exposure* | *Total EDI score*  *b-coefficient (95% CI)* |
| --- | --- |
| Traffic environment domain score | |
| Quartile 2 | 0.33 (0.10, 0.55) |
| Quartile 3 | 0.34 (0.11, 0.57) |
| Quartile 4 | 0.52 (0.26, 0.77) |
| Spaces for play domain score | |
| Quartile 2 | -0.52 (-0.75, -0.29) |
| Quartile 3 | -0.36 (-0.59, -0.13) |
| Quartile 4 | -0.43 (-0.68, -0.18) |
| Social environment domain score | |
| Quartile 2 | 0.24 (0.01, 0.48) |
| Quartile 3 | 0.03 (-0.22, 0.28) |
| Quartile 4 | 0.22 (-0.05, 0.48) |
| Natural environment domain score | |
| Quartile 2 | 0.48 (0.24, 0.72) |
| Quartile 3 | 0.75 (0.48, 1.02) |
| Quartile 4 | 0.83 (0.53, 1.14) |
| Child-relevant destinations domain score | |
| Quartile 2 | -0.05 (-0.28, 0.18) |
| Quartile 3 | -0.20 (-0.44, 0.04) |
| Quartile 4 | -0.32 (-0.58, -0.06) |

Data presented are b-coefficient (95% confidence interval) for each quartile of exposure (reference = quartile 1).

Adjusted models control for age at time of EDI assessment, sex, English as a second language, lone parent household, maternal age, Medical Services Plan (MSP) subsidy, neighborhood-level material deprivation, and neighborhood-level residential instability. Models include random effect for teacher ID.

All estimated effects and 95% confidence intervals are rounded.

EDI, Early Development Instrument.

**Table S5**. Adjusted associations between neighborhood playability and domain scores of early childhood development (range: 0-10) among Metro Vancouver children (n= 30,126).

| *Exposure* | *Physical health and well-being*  *b-coefficient (95% CI)* | *Social competence*  *b-coefficient (95% CI)* | *Emotional maturity*  *b-coefficient (95% CI)* | *Language and cognitive development*  *b-coefficient (95% CI)* | *Communication skills and general knowledge*  *b-coefficient (95% CI)* |
| --- | --- | --- | --- | --- | --- |
| PS |  |  |  |  |  |
| Quartile 2 | 0.01 (-0.04, 0.05) | -0.02 (-0.08, 0.04) | 0.00 (-0.04, 0.05) | -0.06 (-0.12, -0.01) | -0.04 (-0.12, 0.04) |
| Quartile 3 | 0.05 (0.00, 0.09) | 0.07 (0.01, 0.13) | 0.05 (0.00, 0.10) | 0.01 (-0.05, 0.07) | 0.06 (-0.02, 0.14) |
| Quartile 4 | 0.06 (0.02, 0.11) | 0.06 (0.00, 0.12) | 0.03 (-0.02, 0.09) | 0.02 (-0.04, 0.08) | 0.11 (0.03, 0.20) |
| TED |  |  |  |  |  |
| Quartile 2 | 0.04 (0.00, 0.09) | 0.09 (0.03, 0.15) | 0.04 (-0.01, 0.09) | 0.05 (-0.01, 0.10) | 0.11 (0.03, 0.18) |
| Quartile 3 | 0.03 (-0.02, 0.07) | 0.06 (0.00, 0.12) | 0.02 (-0.03, 0.06) | 0.02 (-0.04, 0.07) | 0.12 (0.04, 0.20) |
| Quartile 4 | 0.03 (-0.01, 0.08) | 0.10 (0.03, 0.16) | 0.04 (-0.01, 0.09) | 0.04 (-0.02, 0.10) | 0.11 (0.02, 0.19) |
| SFP |  |  |  |  |  |
| Quartile 2 | -0.06 (-0.11, -0.02) | -0.08 (-0.14, -0.02) | -0.06 (-0.11, -0.01) | -0.09 (-0.15, -0.04) | -0.09 (-0.17, -0.02) |
| Quartile 3 | -0.01 (-0.05, 0.03) | -0.01 (-0.07, 0.05) | 0.01 (-0.04, 0.06) | -0.10 (-0.16, -0.05) | -0.07 (-0.15, 0.00) |
| Quartile 4 | -0.00 (-0.05, 0.04) | -0.05 (-0.11, 0.01) | -0.04 (-0.09, 0.01) | -0.10 (-0.16, -0.04) | 0.00 (-0.08, 0.08) |
| SED |  |  |  |  |  |
| Quartile 2 | 0.05 (0.01, 0.10) | 0.06 (-0.00, 0.12) | 0.02 (-0.03, 0.07) | 0.06 (0.00, 0.12) | 0.12 (0.04, 0.20) |
| Quartile 3 | 0.04 (-0.01, 0.09) | 0.04 (-0.02, 0.11) | 0.01 (-0.04, 0.06) | 0.03 (-0.03, 0.09) | 0.03 (-0.06, 0.11) |
| Quartile 4 | 0.06 (0.01, 0.11) | 0.07 (0.01, 0.14) | 0.03 (-0.03, 0.08) | 0.06 (-0.01, 0.12) | 0.12 (0.03, 0.21) |
| NED |  |  |  |  |  |
| Quartile 2 | 0.08 (0.04, 0.13) | 0.11 (0.05, 0.17) | 0.07 (0.02, 0.12) | 0.02 (-0.03, 0.08) | 0.17 (0.09, 0.25) |
| Quartile 3 | 0.12 (0.07, 0.17) | 0.16 (0.09, 0.22) | 0.13 (0.08, 0.19) | 0.04 (-0.02, 0.11) | 0.25 (0.16, 0.33) |
| Quartile 4 | 0.15 (0.09, 0.20) | 0.18 (0.11, 0.26) | 0.13 (0.08, 0.19) | 0.08 (0.02, 0.15) | 0.23 (0.13, 0.33) |
| CRD |  |  |  |  |  |
| Quartile 2 | -0.01 (-0.05, 0.04) | -0.01 (-0.07, 0.04) | -0.00 (-0.05, 0.04) | -0.02 (-0.08, 0.03) | 0.01 (-0.07, 0.09) |
| Quartile 3 | -0.06 (-0.10, -0.01) | -0.03 (-0.09, 0.03) | -0.00 (-0.05, 0.05) | -0.07 (-0.13, -0.01) | -0.06 (-0.14, 0.02) |
| Quartile 4 | -0.08 (-0.13, -0.03) | -0.09 (-0.15, -0.02) | -0.05 (-0.10, -0.00) | -0.08 (-0.14, -0.02) | -0.10 (-0.19. -0.02) |

Data presented are b-coefficient (95% confidence interval) for each quartile of exposure (reference = quartile 1).

Adjusted models control for age at time of EDI assessment, sex, English as a second language, lone parent household, maternal age, Medical Services Plan (MSP) subsidy, neighborhood-level material deprivation, and neighborhood-level residential instability. Models include random effect for teacher ID.

All estimated effects and 95% confidence intervals are rounded.

PS, composite playability score; TED, traffic environment domain score; SFP, spaces for play domain score; SED, social environment domain score; NED, natural environment domain score; CRD, child-relevant destinations domain score.

**Table S6**. Unadjusted and adjusted associations between neighborhood playability and overall EDI vulnerability among Metro Vancouver children (n= 30,126).

| *Exposure* | *Total EDI vulnerability*  *Odds ratio (95% CI)* | |
| --- | --- | --- |
|  | *Unadjusted* | *Adjusted* |
| Composite playability score | | |
| Quartile 2 | 0.94 (0.87, 1.01) | 1.04 (0.96, 1.12) |
| Quartile 3 | 0.81 (0.75, 0.87) | 0.95 (0.88, 1.03) |
| Quartile 4 | 0.70 (0.64, 0.76) | 0.90 (0.82, 0.98) |
| Traffic environment domain score | | |
| Quartile 2 | 0.97 (0.90, 1.05) | 0.94 (0.87, 1.02) |
| Quartile 3 | 1.01 (0.93, 1.09) | 0.96 (0.88, 1.04) |
| Quartile 4 | 1.01 (0.93, 1.09) | 0.95 (0.87, 1.03) |
| Spaces for play domain score | | |
| Quartile 2 | 1.06 (0.98, 1.14) | 1.09 (1.01, 1.18) |
| Quartile 3 | 0.99 (0.91, 1.06) | 1.04 (0.96, 1.13) |
| Quartile 4 | 0.93 (0.86, 1.00) | 1.08 (0.99, 1.17) |
| Social environment domain score | | |
| Quartile 2 | 0.85 (0.79, 0.92) | 0.91 (0.84, 0.99) |
| Quartile 3 | 0.80 (0.74, 0.86) | 0.93 (0.85, 1.01) |
| Quartile 4 | 0.69 (0.64, 0.75) | 0.89 (0.81, 0.97) |
| Natural environment domain score | | |
| Quartile 2 | 0.81 (0.75, 0.87) | 0.94 (0.87, 1.02) |
| Quartile 3 | 0.62 (0.57, 0.67) | 0.81 (0.74, 0.88) |
| Quartile 4 | 0.50 (0.46, 0.55) | 0.78 (0.71, 0.86) |
| Child-relevant destinations domain score | | |
| Quartile 2 | 1.14 (1.05, 1.23) | 1.02 (0.93, 1.10) |
| Quartile 3 | 1.28 (1.18, 1.38) | 1.07 (0.98, 1.16) |
| Quartile 4 | 1.41 (1.30, 1.53) | 1.15 (1.06, 1.26) |

Data are odds ratio (95% confidence interval) of developmental vulnerability (reference = no) for each quartile of exposure (reference = quartile 1).

Adjusted models control for age at time of EDI assessment, sex, English as a second language, lone parent household, maternal age, Medical Services Plan (MSP) subsidy, neighborhood-level material deprivation, and neighborhood-level residential instability. Models include random effect for teacher ID.

All estimated effects and 95% confidence intervals are rounded.

EDI, Early Development Instrument.
